# Stereotactic Electroencephalogram Recordings in Temporal Lobectomy Patients Demonstrates the Predictive Value of Interictal Cross-Frequency Correlations: A Retrospective Study

**DOI:** 10.3390/brainsci14030212

**Published:** 2024-02-26

**Authors:** Anish Vinay Sathe, Mahdi Alizadeh, Emily Johannan, Christian Raimondo, Michael Sperling, Ashwini Sharan, Michael Kogan

**Affiliations:** 1 Department of Neurological Surgery, Thomas Jefferson University, Philadelphia, PA 19107, USA; mahdi.alizadeh2@jefferson.edu (M.A.);; 2 Department of Neurology, Thomas Jefferson University, Philadelphia, PA 19107, USA; 3 Department of Neurological Surgery, University of New Mexico, Albuquerque, NM 87106, USA; mikogan@salud.unm.edu

**Keywords:** stereotactic electroencephalography, temporal lobectomy, epilepsy, cross-frequency correlations

## Abstract

Background: Positive correlations between low- and high-frequency spectra from stereotactic electroencephalogram (SEEG) recordings have been implicated in pathological brain activity interictally and have been used for ictal detection in both focal and network models. Objective: We evaluated SEEG signals in patients who ultimately underwent temporal lobectomy to evaluate their utility in seizure localization and prediction of seizure freedom post-resection. Methods: We retrospectively analyzed cross-frequency correlations between beta and high gamma (HG) interictal SEEG signals from 22 patients. We compared signals based on temporal versus extra-temporal locations, seizure-free (SF) versus non-seizure-free (NSF) outcomes, and mesial (M) versus mesial temporal-plus (M+) onset. Results: Positive cross-correlations were increased in temporal areas. NSF patients showed a higher proportion of positive electrodes in temporal areas. SF patients had a greater proportion of significant channels in mesial versus lateral temporal areas. HG/Beta correlations in mesial versus lateral temporal areas predicted seizure freedom better than ictal SEEG seizure onset localization to M or M+ locations. Conclusions: We present preliminary data that local HG/Beta correlations may predict epilepsy focus and surgical outcome and may have utility as adjunct methods to conventional SEEG analysis. Further studies are needed to determine strategies for prospective studies and clinical use.

## 1. Introduction

Epilepsy is among the most common and burdensome neurologic diseases [1]. With one-third of the epilepsy population resistant to anti-epileptic medications, surgical resection or neuromodulation is often recommended as treatment for qualifying candidates [2]. The success of surgical outcomes depends on the accurate identification of interictal spikes and/or epileptogenic zones. Although surgical resection has been shown to be effective in 40–80% of operative candidates, the existence of patients who continue to have seizures following surgical intervention emphasizes the importance of developing improved preoperative localization strategies [3]. Modern invasive studies such as stereo-electroencephalography (SEEG), a minimally invasive extra-operative monitoring method that utilizes intracerebral electrodes to measure ictal/interictal brain activity, offer more precise seizure localization with greater options for surgical seizure control. The widespread application of SEEG used for obtaining a spectrum of presurgical neurophysiological data is further supported by its improved safety profile, with lower rates of complications than other methods of intraoperative monitoring [4].

However, the detection of epileptic zones can be difficult, as most strategies rely on capturing ictal data within a limited window of clinical monitoring [5]. While magnetoencephalography and EEG-fMRI are modalities that may facilitate interictal and ictal localization of the seizure onset zone, they are limited in use for presurgical planning by poor spatial and temporal resolution, respectively [6]. Meanwhile, SEEG demonstrates high temporal and spatial resolution within regions implanted with electrodes based on clinical suspicion for seizure onset. One approach to overcome the difficulty of detecting epileptic zones is to analyze network connections between recorded areas to delineate normal and epileptic areas [7,8]. Network studies make use of the observation that there are patterned topological and functional alterations during interictal and ictal stages [8]. Understanding patient networks in epilepsy resting-state may help identify the seizure onset zone without requiring long-term ictal recordings [7]. Additionally, network-based analysis can integrate anatomical location-based data with functional connectivity-based correlations to create more accurate models of brain connectivity [9]. As such, specific subject-independent features can be used to create generalizable models of brain networks to detect patterns of neuronal excitability [10,11].

Alternatively, local recordings may harbor aberrant properties independent of networks. Prior studies demonstrated that local cross-frequency relationships can identify ictal events as defined by the epileptogenicity index (EI) [12,13]. Measures of EI are determined by the presence of two parameters: high-frequency oscillations (HFOs) and delayed involvement of the structure during the event [13]. HFOs as a marker for the seizure onset zone display transient focal increases in amplitude within 80–500 Hz [14]. Additionally, previous studies have shown a correlation between removal of HFO-generating areas and improved surgical outcomes [15]. Additionally, studies have examined methods analyzing seizure-related features, including HFOs, extracted from individual electrodes to detect seizure foci using interictal recordings [16]. However, the clinical implementation of HFOs has yet to reach full maturity, largely due to limitations in specificity when used in awake patients. The presence of overlapping voluntary high-gamma signals (HG; 70–200 Hz) spanning the HFO bandwidth increases the technical challenge of detecting clinically relevant signals. Cross-frequency analysis is a useful technique for differentiating between overlapping HFOs and HGs [17]. Improved strategies for characterizing signals within this range may help distinguish epileptic zones from normal function in awake patients.

Our prior case series demonstrated that in patients with implanted grids, positive HG/Beta correlations (+HBC) localize to the eventual seizure focus [18]. Unlike negative HG/Beta correlations, which are representative of normal voluntary activity, +HBC signals are independent of any voluntary action [19,20]. In our mentioned prior work, patients with the best surgical outcomes had increased +HBC channels in the resection site, while those with poor outcomes were found to have increased +HBC channels outside the area. Additionally, in patients who underwent temporal lobectomy, better outcomes were related to the degree of localization of +HBC channels to the anterior temporal area [18]. These findings suggest that +HBC may help predict lobectomy outcomes by prospectively identifying the number of non-temporally located +HBC channels.

Network-based approaches have also relied on cross-frequency relationships to detect epilepsy-related changes [12]. However, these changes remain poorly characterized in the interictal state, and the relationship to other events, such as sleep/wake cycle, is unknown. It also remains unknown if a local approach to epilepsy detection is more efficient or efficacious compared to network-based models. Accordingly, centers have begun to move away from monitoring via grid implantation and towards SEEG electrodes. SEEG offers the benefit of recording deeper signals due to the implantation of depth electrodes, allowing channels to sample signals from structures beyond the surface cortical structures detected by grids.

As such, in this study, we aim to further explore the detection of +HBC in SEEG recordings to evaluate their predictive potential in seizure focus detection and surgical planning. We aim to determine whether our method, previously demonstrated in grids, translates to SEEG analysis. Further, we used clinically derived recordings without special parallel capture. These were the same recordings used by the neurologists to review the signal. We performed cross-frequency signal processing of beta and HG signals using similar methods to our previous studies. Signals were separated based on temporal (TL) and non-temporal (nTL) implanted electrodes, and mesial (MTL) and lateral (LTL) portions of TL electrodes in patients to broaden the clinical investigation. Lastly, we aim to determine strategies for predicting outcomes in resection patients by analyzing +HBC signals between seizure-free (SF) and not-seizure-free (NSF) groups and patients with mesial (M) and mesial temporal-plus (M+) onset.

## 2. Materials and Methods

This study was approved by IRB# 08F.464R from the Thomas Jefferson University institutional review board on 5 May 2014, with annual renewal for continued approval. The use of patient information from human participants in this study was reviewed and approved by the Ethics Committee of Thomas Jefferson University. The patients/participants provided their written informed consent for their data to be used in retrospective studies. Patient health information used in this study was de-identified after extracting clinical data from the electronic medical record. We retrospectively reviewed all SEEG implants at Thomas Jefferson University between 2 December 2014 and 8 December 2020. Of these, we selected a relatively homogenous population of patients who underwent both SEEG and temporal lobectomy. We were able to obtain signal data for 22 patients, each of whom had been diagnosed with temporal lobe epilepsy (TLE) by an epileptologist. These patients represent a series of consecutive cases in which the patient received SEEG followed by anterior temporal lobectomy (ATL) for TLE. Inclusion criteria for the patient cohort used in this study were sEEG implantation followed by subsequent temporal lobectomy for treatment of medically refractory epilepsy. Other inclusion criteria included the acquisition of presurgical MRI for stereotactic electrode placement and post-implantation CT scans to localize contacts within the brain. Exclusion criteria included the lack of a sufficient (>1 year) clinical follow-up, less than 10 min of available artifact-free clinical recordings taken from sEEG channels, or concurrent treatment of epilepsy via neuromodulation such as responsive neurostimulation, vagal nerve stimulation, or deep brain stimulation for epilepsy. Patients who received temporal lobectomy without prior SEEG implantation as well as patients who received SEEG after temporal lobectomy were excluded. These patients were classified as SF (*n* = 9) if they had no seizures or only had auras within 12 months after receiving surgery (ILAE Class 1 or 2), or NSF (*n* = 13) if they had a seizure within 12 months (ILAE Class 3 or above). Patients were also separately classified in the M cohort if they had a seizure onset zone (SOZ) purely in the mesial temporal lobe (MTL, *n* = 15) or in the M+ cohort if they also had evidence of SOZ in extra-mesial regions, such as the temporal neocortex or the insula (*n* = 7). As the MTL was designated via splitting of temporal electrodes into medial and lateral sections, our classification of the MTL included recordings from any temporal regions located anatomically in the medial aspect of the temporal lobe. This would potentially include structures such as the hippocampus, amygdala, parahippocampal gyrus, dentate gyrus, and others. The locations of the SOZ were determined by a clinical epileptologist using ictal SEEG recordings. Post-implantation high-resolution CT scans were reviewed to ensure that all contacts through which signal channels were obtained were intracranial. We acquired interictal recordings during Day 1, Day 5 daytime awake periods, and Day 5 asleep periods, which were confirmed with patient appearance on video recording. All Day 1 recordings were taken while the patient was awake. One patient had no recordings during the Day 5 asleep period, so Day 2 recordings at night were used instead. The recordings were reviewed by a neurologist to ensure the signals were obtained in an interictal state. The asleep signals were reviewed by a neurologist to determine sleep status. While the exact phase of sleep could not be determined from our limited recordings, we were able to clinically support that the patients were in a sleep state based on the recordings. We utilized night-time recordings of patients with an extended period without movement. These recordings were 30 min in length; we utilized the middle 10 min in our analysis.

We recorded all the studies using the Nihon Kohden recording system between 1000 and 2000 Hz and reviewed them in a bipolar montage. All the raw EEG data are obtained using a digital reference which is equal to zero. The sensitivity ranges from 50 μV to 150 μV with the mean usually being 75 μV. The high pass filter is then set at 1 Hz, and the low pass filter is set at 300–600 Hz, depending on initial recording frequency.

**Figure 1:**
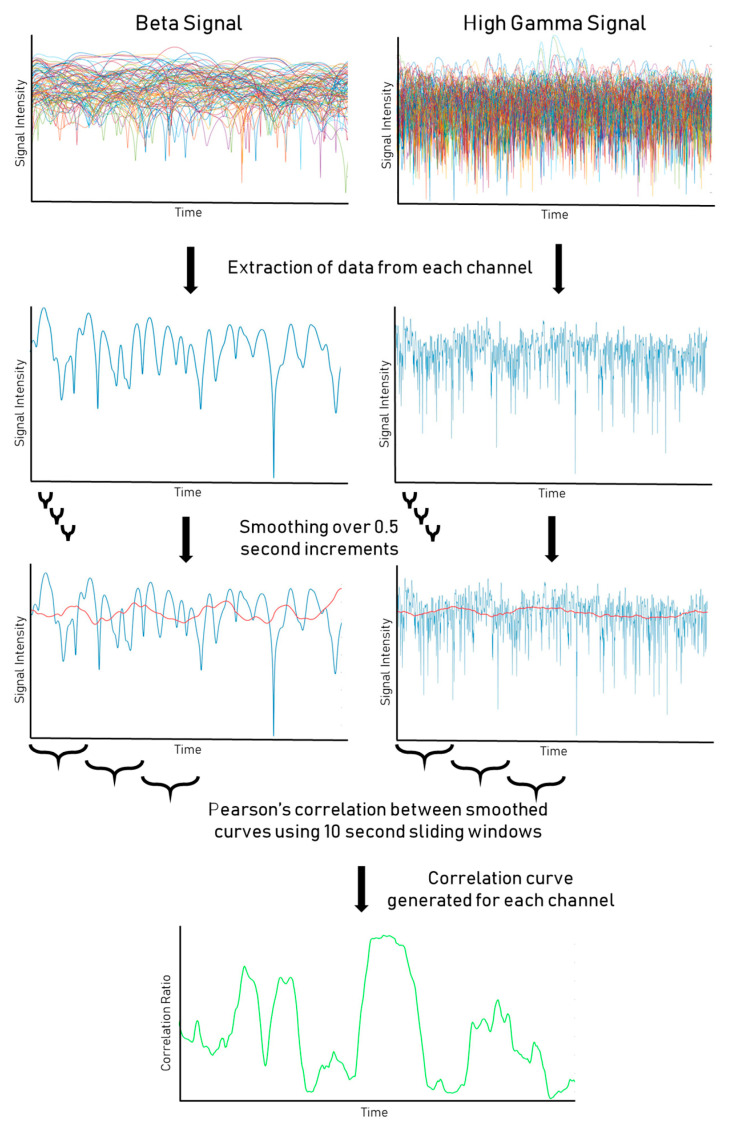
Processing pipeline for SEEG data. HG and Beta signals from SEEG recording are displayed, pre- and post smoothing protocol. Correlation windows between smoothed HG/Beta signals are then calculated to assess the level of significance of the correlations.

Our methodology of +HBC was detailed in our prior publication [18]. We computed power–power correlations using a 4th order Butterworth to bandpass signals to corresponding HG (70–200 Hz) and Beta 1 (12–18 Hz) frequency bands for each electrode site and estimated log power (e.g., amplitude envelope) by computing the natural logarithm of the square of the analytical amplitude of the Hilbert transform [21]. We then smoothed the estimated amplitude envelope using a Gaussian filter transform with a sliding window mean. Our signals were acquired with some patients at 1000 Hz (*n* = 6) and some at 2000 Hz (*n* = 16), due to improved recording capabilities at our institution over the period studied. Smoothing windows used a duration of 0.5 s (Figure 1). The smoothed HG and Beta power estimates were then correlated using Pearson’s correlation using a staggered window of 10 s (Figure 1). This window was advanced stepwise from the first sample to the length of the total signal, with each step being the same length as the window.

The resulting correlations were then calculated for the length of the signal (Figure 1). A global correlation throughout the signal was also calculated to generate the global correlation coefficient for each channel. A one-way one-sample *t*-test was performed on the distribution of correlation coefficients between windows for each channel to calculate a *p*-value. We used an initial alpha of 0.05 and a global correlation coefficient of 0.4 as a cut-off for significance. However, for the resting-state, *p*-values were also adjusted for false discovery rate (FDR) to correct for false positives resulting from the large number of t-tests comparisons [22]. The distribution of significant and nonsignificant channels in an example patient is shown in Figure 1.

Contacts through which channels were obtained were classified as located in the temporal lobe (TL) or outside (nTL), based on a clinical review of the locations of implantation by a neurosurgeon. The ratio of significant to nonsignificant channels was calculated in the TL and nTL regions for each patient. The use of the ratio controlled for differences in the absolute number of channels used in each patient. These ratios were compared for all days combined and for each recorded day using nonparametric matched-pairs Wilcoxon tests. Patient recordings were also compared between TL and nTL regions both in SF patients and in NSF patients, separately, using Wilcoxon tests. The ratios were also compared between SF and NSF patients in TL and in nTL channels, separately, using nonparametric Mann–Whitney tests. Significant values were reported with an alpha of 0.05, adjusted to 0.013 using Bonferroni correction to account for the multiple comparisons problem. Nonparametric tests were used for comparison as the data were not found to pass tests of normality.

Electrodes situated in the TL were further analyzed by separating channels on each electrode into halves representing the MTL and lateral temporal lobe (LTL). If an electrode contained an odd number of channels, the middle channel was discarded. The ratio of significant channels in the MTL to significant channels in the LTL was compared between SF and NSF patient cohorts as well as between M and M+ onset cohorts using nonparametric Mann–Whitney tests. Significant values were reported with an alpha of 0.05. Patients were also classified on an individual basis as whether they had more significant MTL leads than LTL or not, and odds ratios were constructed between SF and NSF cohorts as well as between M onset and M+ onset cohorts. A threshold was also calculated based on the mean ratio of significant channels in SF and NSF patients to test how well the value could separate SF and NSF patients. The sensitivity, specificity, positive predictive value (PPV), and negative predictive value (NPV) of this threshold test were calculated.

## 3. Results

We analyzed 22 patients who received SEEG implantation and eventual temporal lobectomy for medically refractory epilepsy. Patient characteristics, treatment history, and surgical outcomes are described in Table 1 and Table 2.

**Figure 2:**
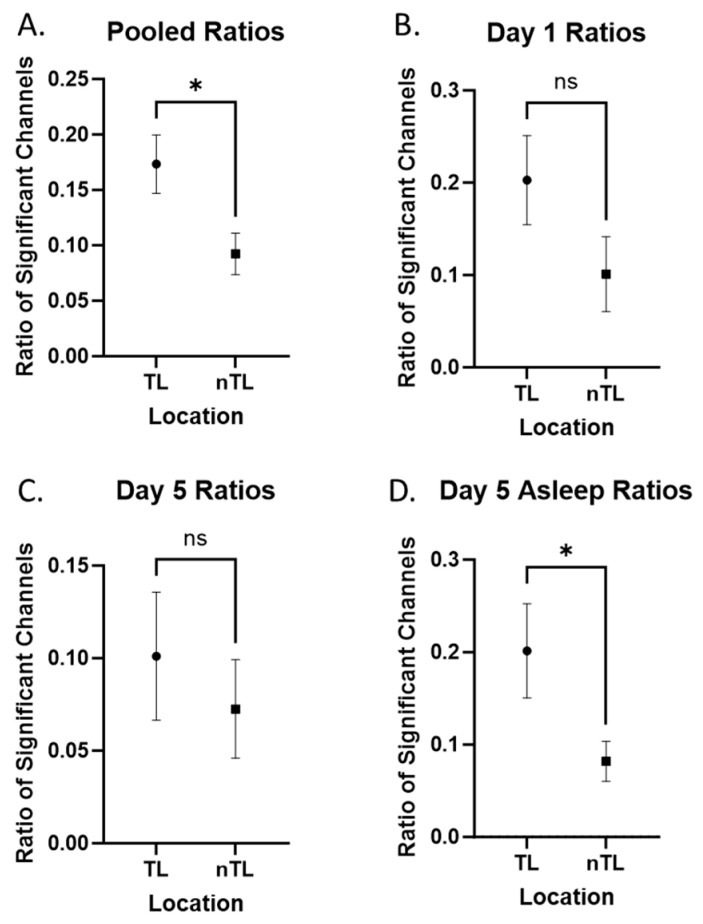
Temporal versus non-temporal signals. Comparisons labeled with “ns” are not statistically significant. Comparisons labeled with “*” are statistically significant at an alpha of 0.05. (**A**) Pooled ratios (day 1, day 5, day 5 asleep) comparing tagged electrodes from TL and nTL areas (contralateral temporal lobe included with non-temporal areas). (**B**–**D**) Day 1, day 5, and day 5 signals individually compared between TL and nTL areas.

We first evaluated our pooled data by comparing the ratio of significant +HBC during 10 min of recording from day 1, 5, and 5 asleep between channels in TL and nTL regions (Figure 2A). TL structures showed significantly higher +HBC ratios (*p* = 0.013). When separating these signals into respective days, we could not demonstrate the same findings for Day 1 or Day 5 while awake, although the findings for Day 1 showed the same trend (*p* = 0.068, Figure 2B; *p* = 0.571, Figure 2C). Asleep signals on Day 5 were more robust compared to Day 5 awake signals, and once again demonstrated increased presence in the TL region (*p* = 0.009, Figure 2D). In all cases, there was an increase in +HBC in temporal areas. The mean ratios, standard deviations, significance, and effect sizes are reported in Table 3.

**Figure 3:**
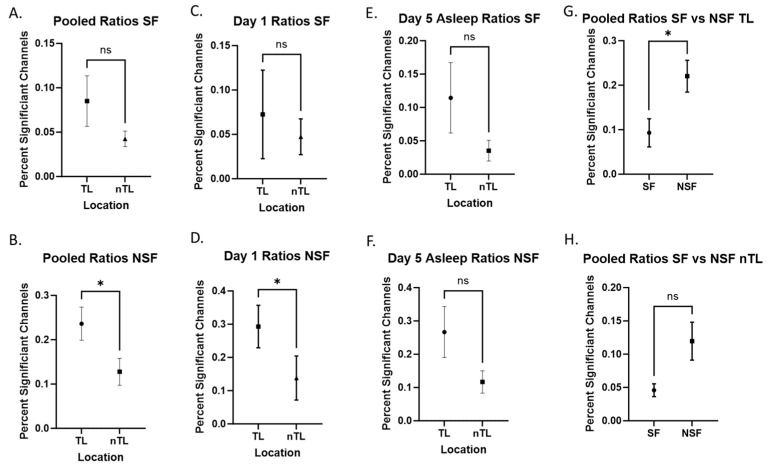
Seizure-free versus non-seizure-free. Comparisons labeled with “ns” are not statistically significant. Comparisons labeled with “*” are statistically significant at an alpha of 0.05. (**A**,**B**) Combined ratios comparing SF and NSF patients. (**C**,**D**) Day 1 comparing SF and NSF patients. (**E**,**F**) Day 5 asleep comparing SF and NSF patients. (**E**,**F**) SF vs. NSF directly compared looking at only TL (**G**) and nTL (**H**) electrodes.

We then analyzed patients based on their seizure outcomes. We evaluated signals in these patients during pooled, Day 1, and Day 5 asleep recordings, as these were most significant in our previous analysis. Our data show that NSF patients showed a significantly increased ratio of +HBC in TL compared to nTL in pooled (*p* = 0.012, Figure 3B) recordings, but not in Day 1 (*p* = 0.048, Figure 3D) and Day 5 asleep (*p* = 0.052, Figure 3F), although these showed similar trends. Meanwhile, SF patients did not show significant differences in recordings in pooled (*p* = 0.600, Figure 3A), Day 1 (*p* = 1.000, Figure 3C), or Day 5 asleep (*p* = 0.148, Figure 3E) recordings. Comparing SF and NSF patients directly, we found TL areas had significantly more +HBC in NSF patients (*p* = 0.003, Figure 3G). Looking at nTL areas, there was no significant difference between SF and NSF patients, although there was also a trend of increased +HBC in the NSF group (*p* = 0.064, Figure 3H). The means and standard deviations of these data are represented in Table 4.

**Figure 4:**
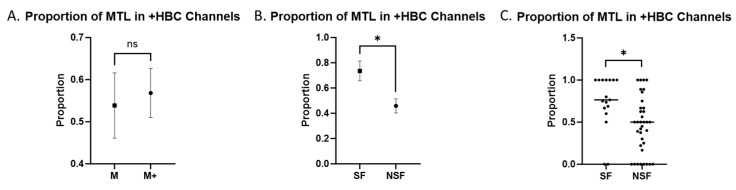
MTL vs. LTL +HBC Channels. Combined ratios comparing the proportion of +HBC channels located in the MTL to all TL +HBC channels. Comparisons labeled with “ns” are not statistically significant. Comparisons labeled with “*” are statistically significant at an alpha of 0.05. (**A**) Mean ± SEM in M versus M+ onset patients. (**B**) Mean ± SEM in SF versus NSF patients grouped together. (**C**) Individual values with the median in SF versus NSF patients.

We also compared the number of significant TL channels found in the MTL and LTL in patients, based on seizure outcomes and on seizure onset region. We found no significant correlation between M onset and SF after resection (odds ratio = 0.89, CI = 0.1565 to 4.574, *p* = 1.00). Patients in the M onset cohort did not show a significant difference in the MTL-to-total significant channel ratio compared to the M+ onset cohort (*p* = 0.707, Figure 4A). Meanwhile, patients in the SF cohort had a higher ratio of MTL to total significant channels compared to the NSF cohort (*p* = 0.003, Figure 4B). Two data points in the SF cohort and 16 in the NSF cohort showed an MTL-to-total ratio of significant channels of less than 0.5 in recordings from any day (Figure 4C). The odds ratio for SF patients to have more significant MTL than LTL channels compared to NSF patients was 1.988 (95% CI = 0.6932 to 5.207, *p* = 0.208). The odds ratio for M onset patients to have more significant MTL than LTL channels compared to M+ onset patients was 0.741 (95% CI = 0.2506 to 2.251, *p* = 0.594). The means and standard deviations of each group are displayed in Table 5.

The calculated threshold between the SF and NSF patients was 0.597. The data were analyzed based on whether this value could effectively separate SF and NSF patients. The sensitivity of this test was 0.824, the specificity was 0.667, the PPV was 0.538, and the NPV was 0.889 (Table 6).

## 4. Discussion

Our results confirm the consistency of the findings of our previous study examining invasive grid recordings to SEEG recordings in this study. Mainly, we re-demonstrate that +HBC localized to seizure onset areas in the TLE patient population. This study analyzed SEEG recordings, rather than subdural grid recordings as used prior, to determine whether this methodology can be translated to SEEG analysis. We were able to demonstrate statistical significance in eventually resected TL areas compared to nTL, both in pooled and non-pooled samples.

Interestingly, we found that these signals decreased during extended monitoring, but increased during asleep periods. Although the difference between TL and nTL based on the Day 1 recordings was not significant, the near significance may warrant further study with a larger sample size. The decrease in significance of +HBC from Day 1 to Day 5 in awake recordings may suggest that these signals diminish over the course of phase II monitoring. When comparing Day 5 awake and asleep signals, it appears that these signals increase during asleep recordings. Multiple factors may influence the change in these signals during interictal periods. This includes proximity to ictal events or postictal periods, recovery from anesthesia, and decrease in antiepileptic drugs. Furthermore, the presence of these signals increases at night, corresponding to known findings of nocturnal changes in epilepsy [23]. Similarly, previous studies have observed higher ratios of abnormal sleep EEGs in patients with focal epilepsy and abnormal sleep EEGs in 41.8% of epilepsy patients with normal awake EEGs [24]. In our study, we were not able to retrospectively characterize the sleep phase and could only establish that the patient appeared to be asleep during night-time hours. We used the middle 10 min segments of 30 min recordings acquired at night during which patients showed a lack of movement. However, it is possible that patients were rather in an awake resting state, and some of our recordings classified as asleep may include awake, resting-state signals. As such, differences in +HBC that we found in Day 5 awake and asleep recordings may partially reflect differences in activated versus resting-state awake states rather than awake and asleep or may be a result of differences in brain signaling over the course of the day and night. Future studies may trend the presence of these signals during phase II monitoring to evaluate what factors and stages of sleep influence the presence of interictal +HBC. However, few strategies exist to detect the sleep state in SEEG [25]. A hybrid approach with scalp electrodes may be worthwhile in the future [26]. Although we did not investigate the relationship between age and +HBC channels, future studies may benefit from this consideration due to prior evidence of a correlation between younger age and abnormal sleep EEG [24].

We also grouped patients based on their seizure onset regions and seizure outcomes after resection. In our study population, we found similar rates of seizure freedom in patients with M and M+ onset. Prior literature has shown that M+ and extra-TL onset of seizures is associated with poorer surgical outcomes [27,28]. The lack of correlation between onset region and seizure outcomes in our study may be due to the smaller sample size of the study. However, the patients grouped in the M+ cohort had a component of MTL onset along with other extra-TL seizure onset regions; resection of the MTL component of the SOZ or interruption of a seizure network may have been sufficient for the patient to become SF.

We showed for the first time that +HBC may predict not only seizure onset, but outcome after resection, with a higher proportion of +HBC channels in the TL predicting poor surgical outcomes. To our surprise, increased +HBC signals were noted in TL rather than nTL areas in NSF patients. While we hypothesized that nTL areas would have an increased ratio compared to TL areas, we did note that both areas had a relative increase in the NSF patients, although this was not significant in the nTL areas. There could be multiple reasons for this finding. For one, our TL area was defined by the anatomic location of the electrodes, not the SOZ or ultimate resection area. It is possible that posterior temporal electrodes, while positive, were not within the resection cavity, particularly on the left side. Indeed, in temporal lobectomy failures, posterior mesial and lateral temporal areas account for significant cause of relapse [29]. Further analysis of channels based on their placement in regions of seizure onset versus spread versus uninvolved areas may be useful. Using the findings of this study as a proof-of-concept foundation, we aim to explore specific volume and imaging-based correlations with seizure outcomes in future studies. While these data are preliminary, further studies correlating the +HBC method of analysis we explored in this study with a three-dimensional reconstruction of electrode location and co-registration with post-resection scans may yield a more accurate prediction of seizure freedom by looking at positive electrodes, specifically in resected areas.

To investigate differences in correlations based on subsections within the TL, we divided electrodes located in the TL into mesial and lateral halves. While this method of division of channels into mesial and lateral halves is not perfect, we utilize this as an approximation of the regions sampled by our TL electrodes. As we know the trajectory of the electrodes is orthogonal to the surface cortex, we can say that the more proximal sections sample more LTL while the more distal channels sample more MTL. The channels closer to the middle were likely traveling through white matter regions, which are generally silent, therefore forming a buffer region to properly separate the MTL and LTL signals. Given that +HBC likely represents regional differences, this approach is likely a useful characterization of the distribution of these signals within the temporal lobe. Using these sections, we compared correlations in the MTL and LTL between patients with M and M+ onset and between the SF and NSF cohorts. We found that patients in the SF cohort had significantly higher proportions of channels showing +HBC in the MTL vs. the LTL compared to patients in the NSF cohort. This provides further support for the idea that +HBC localizes to seizure onset regions, as the localization of +HBC to the resected MTL portion of the TL provides better seizure outcomes. In addition, we did not find a similar significant correlation in the ratio of MTL to LTL +HBC channels between M versus M+ onset as determined by ictal SEEG recordings, indicating that +HBC channels may represent pathologic, interictal seizure activity different from that detected by ictal SEEG recordings. Even more interestingly, only two of the recordings in the SF cohort showed a ratio of MTL-to-total significant channels of less than 0.5, while the NSF cohort had 16 such recordings. Additionally, the odds ratio calculation showed that patients with more +HBC channels in the MTL than the LTL had nearly double the likelihood of being in the SF cohort, although this was not significant. The 2 × 2 table analysis based on these results showed a fairly high sensitivity but low specificity for predicting patient outcomes after ATL. These findings raise the possibility of using the proportion of MTL +HBC channels as a screening tool or prognostic biomarker to predict whether patients will respond well to epilepsy surgery, as those with an MTL-to-total +HBC ratio of less than a threshold value of around 0.5–0.6 may be considered poor candidates. Prior studies have examined different interictal signal features as potential biomarkers to identify epileptogenic regions. For example, resection of regions generating interictal HFO signals has been shown to be associated with better surgical outcomes [15,30]. Ren et al. found gamma oscillations preceding interictal epileptiform activity were associated with the SOZ [31]. Shamas et al. (2022) found that SF patients demonstrated greater event connectivity within the SOZ in the low gamma and HG frequency bands compared to NSF patients [32]. Other studies demonstrated the importance of resecting areas with high onset-ripple rates in pediatric patients with epilepsy for improved outcomes [33]. Furthermore, these results support the trend towards non-invasive approaches, which have been demonstrated in recent studies that have used non-invasive HD-EEG and MEG to map epileptogenic networks and identify critical network hubs for predicting epileptic zones and surgical outcomes in patients with DRE [34]. These different signal features may all represent various methods to detect abnormal interictal activity located within seizure-related regions. Indeed, our method of +HBC analysis may be processing similar features to HFOs and HG interictal signals while minimizing noise from overlapping signals. We were unable to adequately measure HFO signals due to technical limitations in the measurement of especially high-frequency signals, as we would require higher temporal sampling resolution [35]. However, future studies using recordings with more robust temporal resolution may use these various interictal biomarkers with our method of +HBC analysis to compare their effectiveness in localizing the SOZ.

While these are still preliminary results, the findings of this study provide further support for the clinical application of +HBC signaling to aid preoperative planning for surgical resection in medically refractory TLE as an adjunct method of analysis. While TL resection is known to mitigate seizure symptoms, the degree of success remains variable. Previous reports have shown a success rate of 70% of patients achieving freedom from disabling seizures with about 58% free from seizures altogether [36]. Even when the SOZ as determined via conventional SEEG analysis is resected, seizure-free rates have been shown to range from around 60 to 80%, showing that the current method of SEEG analysis for the detection of the SOZ may be improved [29,37,38]. In this analysis, we have about a 40% SF rate, but many of these are non-lesional cases. The success of resection depends on the degree of elimination of seizure symptoms, considering that quality-of-life scores deteriorate with less than 90% symptom reduction [39]. Although repeat surgical procedures may remain an option for patients experiencing refractory seizures following an initial surgical resection, additional treatment increases the burden and cost of healthcare services [40,41]. Further validation of +HBC correlation with ictal onset areas and epileptic zones may aid in the adoption of cross-frequency strategies and improve the effectiveness of surgical resection.

### Limitations and Future Directions

A limitation of this study is the degree to which we could localize the SEEG electrodes. While we could compare TL with nTL channels, we were unable to incorporate more detailed location data in our analysis. The TL covers a large area, much of which may be relatively uninvolved in seizure generation and/or propagation. Regardless, we were aiming to explore our method of correlation analysis as a proof-of-concept study to find alterations in broad regions rather than in specific, focused seizure onset zones to determine whether +HBC analysis could effectively detect regional signal alterations and separate patients based on response to surgery. Future studies with detailed electrode localization data may be able to discern differences in correlations within more specific, physiologically relevant sub-regions and more specific SOZ areas. We aim to eventually utilize co-registration of post-surgical imaging with SEEG localization to determine whether this analysis can effectively predict the location of channels in the ultimately resected area, and whether this correlates with seizure freedom. A challenge of such post-resection imaging correlation is deformation of the surgical cavity and atrophy/shape alteration of temporal structures postoperatively [42,43], creating difficulty in pinpointing the exact contact location to the cavity. As such, we aim to provide a proof-of-concept study to preliminarily support this method of analysis prior to applying it to more difficult clinical–surgical correlations.

Additionally, when looking at patient recordings, we had no data on Day 1 asleep signals. As such, while we found that Day 5 asleep recordings showed increased correlations, we were unable to ascertain the same with Day 1 asleep recordings. Future studies may be performed to test the effectiveness of recordings at different time points and with the patient in different neurological states to better determine signal and correlation detection. We were also unable to definitively categorize recordings as being taken during the patients’ sleep state or determine the exact sleep stage of patients using our SEEG recordings. Obtaining simultaneous intracranial and scalp EEG recordings in future studies may be able to better categorize SEEG signal analysis using sleep stage.

Additionally, the analysis of +HBC channels in the MTL and LTL utilized all sets of recordings from each patient to increase the study power. This may bias study results by pulling data from the same patient multiple times. However, each recording was performed independently and at different time points, decreasing the risk of data being repeated. Furthermore, the goal of this study was to provide initial data to examine possible correlations with +HBC, localization, and seizure outcomes, and as such, methods to increase study power were used. Future studies with greater sample sizes may utilize recordings from only one, uniform time point for each patient.

Our patient population was fairly homogenous when categorized by epilepsy type and location. Our results are likely applicable to a similar population consisting of patients with TLE and may provide additional data for relevant clinical decision-making in such a group. However, future studies may be necessary to characterize results in populations of patients with specific subtypes of epilepsy, including those with focal epilepsy with different seizure onset regions or focal versus focal-to-bilateral epilepsy. Additionally, while we included patients with both right- and left-sided mesial onset, prior literature has shown that seizure outcomes are not different based on laterality in these patients [44]. While surgical resection may be more difficult in patients with a posterior neocortical left-sided onset, any such patients in our cohort were reviewed by neurologists and neurosurgeons and were determined to be appropriate for such a procedure.

## 5. Conclusions

We present preliminary data showing the potential utility of +HBC analysis as an adjunct method to conventional SEEG analysis in seizure focus localization and surgical prognosis. Our findings demonstrate statistically significant increases of local +HBC in TL seizure onset areas, particularly during night-time asleep interictal recordings. We also show that these signals may be capable of potentially predicting ultimate seizure outcomes after surgery. Further studies are needed to further characterize the variability of these signals, exact location relative to resection areas, and determine how they may be used prospectively for clinical decision-making.

## Figures and Tables

**Table 1:** Patient Characteristics. Age, sex, prior surgery status, lateralization, SF status, onset zone, and epilepsy surgeon for the 22 patients included in the study.

Subject Demographics		
	Total	Percent
Total Patients	22	
Male/Female	(13/9)	
Age (ave)	19–52 (32.8)	
Prior Surgery	4	18
Right/Left	(13/9)	
M+	7	32
SF	9	41
Evidence of MTS	7	32
PET scan done	15	68
Surgeon A/B/C	(14/7/1)	

M+ = mesial-plus temporal lobe epilepsy, SF = seizure-free at 1 year post-resection, MTS = mesial temporal sclerosis, PET = positron emission tomography.

**Table 2:** Seizure type and recording information for each patient included in this study.

Patient	Number of Electrodes	Number of Contacts	M Status	SF Status
1	9	109	M	NSF
2	14	152	M	NSF
3	15	159	M	NSF
4	10	126	M+	NSF
5	15	165	M	SF
6	15	173	M+	SF
7	9	96	M	NSF
8	13	114	M	SF
9	23	224	M+	SF
10	14	156	M+	NSF
11	13	155	M	SF
12	12	133	M+	SF
13	14	162	M	NSF
14	12	103	M	SF
15	17	156	M+	NSF
16	13	153	M	NSF
17	17	165	M	SF
18	20	130	M	NSF
19	9	121	M	NSF
20	16	162	M	SF
21	14	135	M	NSF
22	10	118	M+	NSF

M = pure mesial onset epilepsy, M+ = mesial-plus temporal lobe epilepsy, SF = seizure-free at 1 year post-resection, NSF = not seizure-free at 1 year post-resection.

**Table 3:** Means and standard deviations for the proportion of significant +HBC channels in TL and nTL regions, separated by day of recording.

	Mean TL	SD TL	Mean nTL	SD nTL	*p*-Value	Cohen’s D
Pooled Ratios	0.173	0.213	0.092	0.151	0.013	0.439
Day 1 Ratios	0.203	0.226	0.101	0.190	0.068	0.487
Day 5 Awake Ratios	0.101	0.159	0.073	0.122	0.571	0.201
Day 5 Asleep Ratios	0.201	0.234	0.082	0.100	0.009	0.666

**Table 4:** Mean and standard deviations for the proportion of significant +HBC channels in TL and nTL regions, separated by SF status and day of recording.

	Mean TL	SD TL	Mean nTL	SD nTL	*p*-Value	Cohen’s D
Pooled Ratios SF	0.736	0.322	0.736	0.322	0.600	0.389
Day 1 Ratios SF	0.459	0.332	0.459	0.332	1.000	0.220
Day 5 Awake Ratios SF	0.539	0.338	0.539	0.338	0.426	0.219
Day 5 Asleep Ratios SF	0.568	0.344	0.568	0.344	0.148	0.679
Pooled Ratios NSF	0.736	0.322	0.736	0.322	0.012	0.516
Day 1 Ratios NSF	0.459	0.332	0.459	0.332	0.048	0.659
Day 5 Awake Ratios NSF	0.539	0.338	0.539	0.338	1.000	0.197
Day 5 Asleep Ratios NSF	0.568	0.344	0.568	0.344	0.052	0.733

**Table 5:** Means and standard deviations of the proportion of MTL in +HBC channels in SF, NSF, M, and M+ patients.

	Mean	SD	*p*-Value	Cohen’s D
SF	0.736	0.322	0.003	0.087
NSF	0.459	0.332		
M	0.539	0.338	0.707	0.847
M+	0.568	0.344		

**Table 6:** Sensitivity, specificity, PPV, and NPV calculations for the threshold test between SF and NSF patients.

Statistic	Value
Sensitivity	0.824
Specificity	0.667
PPV	0.538
NPV	0.889

## Data Availability

The raw data used in this study are available upon request to the corresponding author.
